# Genome-wide and gene-based association mapping for rice eating and cooking characteristics and protein content

**DOI:** 10.1038/s41598-017-17347-5

**Published:** 2017-12-08

**Authors:** Xiaoqian Wang, Yunlong Pang, Jian Zhang, Zhichao Wu, Kai Chen, Jauhar Ali, Guoyou Ye, Jianlong Xu, Zhikang Li

**Affiliations:** 1 Institute of Crop Sciences/National Key Facility for Crop Gene Resources and Genetic Improvement, Chinese Academy of Agricultural Sciences, Beijing, 100081 China; 2 State Key Laboratory of Crop Biology, College of Agronomy, Shandong Agricultural University, Taian, 271018 China; 3 Agricultural Genomics Institute, Chinese Academy of Agricultural Sciences, Shenzhen, 518120 China; 4 International Rice Research Institute, DAPO Box 777, Metro Manila, Philippines; 5 Shenzhen Institute of Breeding and Innovation, Chinese Academy of Agricultural Sciences, Shenzhen, 518120 China

## Abstract

Rice eating and cooking quality and protein content (PC) are important properties affecting consumers’ preferences, nutrition and health. Linkage QTL mapping and association studies are usually applied to genetically dissect related traits, which could be further facilitated by high density SNP markers and gene annotation based on reference genome to rapid identify candidate genes associated with interested traits. Here, we carried out an association study for apparent amylose content (AC), gel consistency (GC), gelatinization temperature (GT) and PC evaluated in two environments using a diverse panel of 258 accessions from 3 K Rice Genome Project. Wide phenotypic variations were observed in this panel. Genome-wide association study using 22,488 high quality SNPs identified 19 QTL affecting the four traits. Combining gene-based association study and haplotype analyses plus functional annotation allowed us to shortlist nine candidate genes for four important QTL regions affecting AC, GC and GT, including two cloned genes (*Wx* and *ALK*), and seven novels. The research suggested that GWAS and gene-based association analysis followed by haplotype analysis is an effective way to detect candidate genes. The identified genes and QTL provided valuable sources for future functional characterization and genetic improvement of rice eating and cooking quality and PC.

## Introduction

Rice (*Oryza sativa* L.) is the staple food for more than half of the world’s population. With the improvement of living standards and increases of diverse demands, rice grain quality has become one of the foremost considerations for rice breeders, producers and consumers. Rice grain quality consists primarily of four components: milling, appearance, eating and cooking, and nutritional qualities. The last two are especially important, as they are related to consumers’ preferences, nutrition and health.

Rice eating and cooking quality (ECQ) is determined mainly by three major physicochemical characteristics, namely, apparent amylose content (AC), gel consistency (GC) and gelatinization temperature (GT). AC is demonstrated to be the most important factor affecting rice ECQ^1^. AC can be roughly classified into five levels: waxy (1–2%), very low (5–12%), low (12–20%), intermediate (20–25%) and high (>25%)^2^. Cooked rice kernels with high AC are usually dry, separate, less tender and become hard upon cooling, whereas those with low or intermediate AC are glossy, soft and sticky^3^. Intermediate AC rice is widely preferred in most rice producing areas of the world since this kind of rice is soft but not too sticky^4^. Rice cultivars varying in AC could meet the diverse demands for food products and consumers^5^. GC is a fluid property of rice starch gel, which can be classified into three levels: hard (≤40 mm), medium (41–60 mm) and soft (≥60 mm). Cooked rice with high GC tends to be softer and more elastic. GT is a physical trait responsible for rice cooking time. Usually, GT is estimated as alkali spreading value (ASV) that is assessed by the extent of dispersal of whole milled rice grains in dilute alkali solution (1.7% potassium hydroxide [KOH])^6^, and could be classified into four groups: high (1–2), high-intermediate (3), intermediate (4–5) and low (6–7)^7^. Rice kernels with low or intermediate GT need less cooking time which is a desired trait for high quality rice varieties^8^. As one of the most important parameters of nutritional quality, protein content (PC) is the most abundant constituent in milled rice except starch^2^. With increasing attention on health, rice with distinct nutritional quality is required to meet the special requirements of consumers.

To facilitate rice high quality breeding, dissecting the genetic basis of rice grain ECQ and PC is useful. The pathway of starch synthesis has been comprehensively studied^9^. Among these starch synthesis related genes (SSRGs), *Wx* and *ALK* are major genes governing AC and GC and GT, respectively^1^. Other SSRGs, such as *AGPlar*, *BEI*, *GBSSII*, *GPT1*, *ISA2*, *PUL*, *SSI*, *SSIIb*, *SSIIc*, *SSIIIa*, *SSIIIb* and *SSIVa* also have minor effects^10,11^. However, the pathway of protein synthesis remains unclear to date^12^. QTL mapping is an effective way to dissect the genetic basis of quantitative traits. Many QTL affecting rice ECQ and PC have been identified through linkage mapping^13–16^ and association studies^17–20^, but QTL cloning is still a major challenge to plant geneticists and molecular biologists since the classical strategy using map-based cloning is extremely time-consuming and troublesome^21^. With the development of technology and reduced costs of genotyping, it’s increasingly easy to obtain genotypic data with millions of SNP markers through genotyping by sequencing (GBS) and high density SNP chips^22,23^. The high density SNP markers and gene annotation based on high quality reference genomes powerfully facilitate the identification of QTL candidate genes associated with interested traits^24^. Combining genome-wide association study (GWAS) and gene-based association analysis followed by haplotype analysis is an effective way to identify candidate genes for complex traits including rice grain appearance traits^25^.

Therefore, the objective of our study is to identify candidate genes affecting rice grain ECQ and PC using GWAS, and gene-based association analysis combining haplotype analysis. A diverse panel consisting of 258 accessions selected from 3 K Rice Genome Project (3 K RGP)^26^ was evaluated for rice grain ECQ and PC in two environments. GWAS was performed using genome-wide SNPs generated from 3 K RGP by high-throughput sequencing technologies^27^. Then, for important QTL regions, gene-based association analysis was performed using all available SNP from Rice SNP-Seek Database^28^. Finally, haplotype analysis was conducted and the phenotype differences among major haplotypes were tested by ANOVA. By this way, numbers of candidate genes governing investigated traits were determined.

## Materials and Methods

### Plant materials

The materials used in this study comprised 258 rice accessions having similar heading date selected from the 3 K RGP. The detail accession information was described by Wang, *et al*.^25^. Roughly, these rice accessions mainly included seven types. Most of them were *Xian* (*indica*) (174), followed by *Geng* (*japonica*) including *temperate Geng* (32), *tropical Geng* (24) and *subtropical Geng* (14). The remaining were *admixture* type (7), *aus*/*boro* (3) and *basmati*/*sandri* (4)^25^.

### Field trials and trait measurements

These accessions were grown in two environments, including Sanya (SY) during Dec 2014 – Apr 2015 and Shenzhen (SZ) during Mar – Jul 2015. In both environments, each accession was planted in a two-row plot with 10 individuals in each row at a spacing of 20 cm × 25 cm with two replicates for each accession. At the maturity (about 35 days after flowering), eight uniform plants in the middle of each plot were bulked harvested and air-dried for three months in the drying houses. Then, around 150 g seeds were dehulled in an electrical dehuller (model JLGJ-45, China) and milled by a desk-top rice miller (JNMJ 6, China). The physicochemical quality traits including AC, GC, GT and PC were analyzed by near infrared spectroscopy (NIRS) using Infratec 1241 Grain Analyzer (FOSS, Denmark) equipped with STM model^29,30^. About 60 g head milled rice grains of each accession were scanned in duplicate and the average trait value of each accession was used in the following analyses.

### Genome-wide marker-trait associations

We carried out GWAS to detect marker-trait associations for all measured traits utilizing 22,488 high quality SNPs and the mean grain quality trait values of the 258 accessions in the two environments. All statistical analyses for GWAS were performed using the SVS software package (SNP & Variation Suite, Version 8.4.0). An EMMAX (Efficient Mixed-Model Association eXpedited)^31,32^ implementation of the single-locus mixed linear model was applied to the marker dataset. This mixed linear model (MLM) allowed correction for cryptic relatedness and other fixed effects using kinship matrix (K) and population stratification through principle components (Q). The Bonferroni multiple testing correction was applied to identifying significant markers. Significant SNPs affecting the investigated traits were claimed when the test statistics reached P < 1.0 × 10^−4^ in at least one of the two environments. Our previous study found that the maximum linkage disequilibrium (LD) of the current panel was 0.62^25^, thus significant SNPs on the same chromosome with LD higher than 0.31 (half of its initial value)^25^ were delineated into a single QTL.

### Gene-based association and haplotype analysis

**Figure 1:**
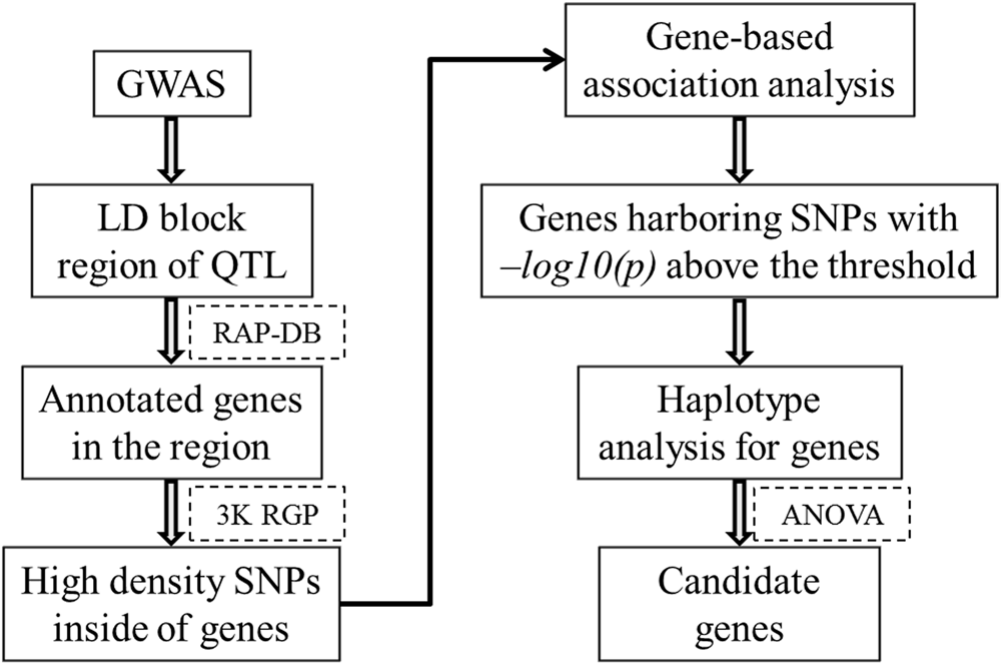
Technical line of combining GWAS and gene-based association analysis to identify candidate genes. RAP-DB: Rice Annotation Project Database. 3 K RGP: 3 K Rice Genome Project.

Gene-based association analysis was carried out to identify candidate genes for important QTL (accounting for over 10% of the trait phenotypic variance). The following four steps were conducted to identify QTL candidate genes. Firstly, we examined all genes located in the 0.31 LD block region of the peak SNP of each important QTL from the Rice Annotation Project Database (RAP-DB). The 0.31 (half of its initial value)^25^ LD block region means, on average, the LD of markers flanking each peak SNP marker is 0.31. In other words, the average LD of each peak marker with its flanking markers across the population is ≥0.31. Statistically, SNPs beyond the 0.31 LD region centered at the peak SNPs are less likely to contain genes responsible for the detected trait-marker association. Then, all available SNPs located inside of these genes were searched from 32 M SNPs data generated from 3 K RGP in the Rice SNP-Seek Database^28^. Thirdly, the SNPs with minor allele frequency less than 0.05 and/or missing rate over 20% were removed and the remaining high quality SNPs were used to perform association analyses through MLM using the Q and K applied in GWAS. The threshold was defined as −log10(p) of peak SNP minus 1; Finally, for the genes harboring SNPs with −log10(p) above the threshold, haplotype analysis was carried out, and candidate genes were determined by testing the significant differences among major haplotypes (containing more than 10 samples) through ANOVA. Figure 1 illustrated the technical line of combining GWAS and gene-based association analysis to identify candidate genes.

## Results

### Trait variations and correlations

**Figure 2:**
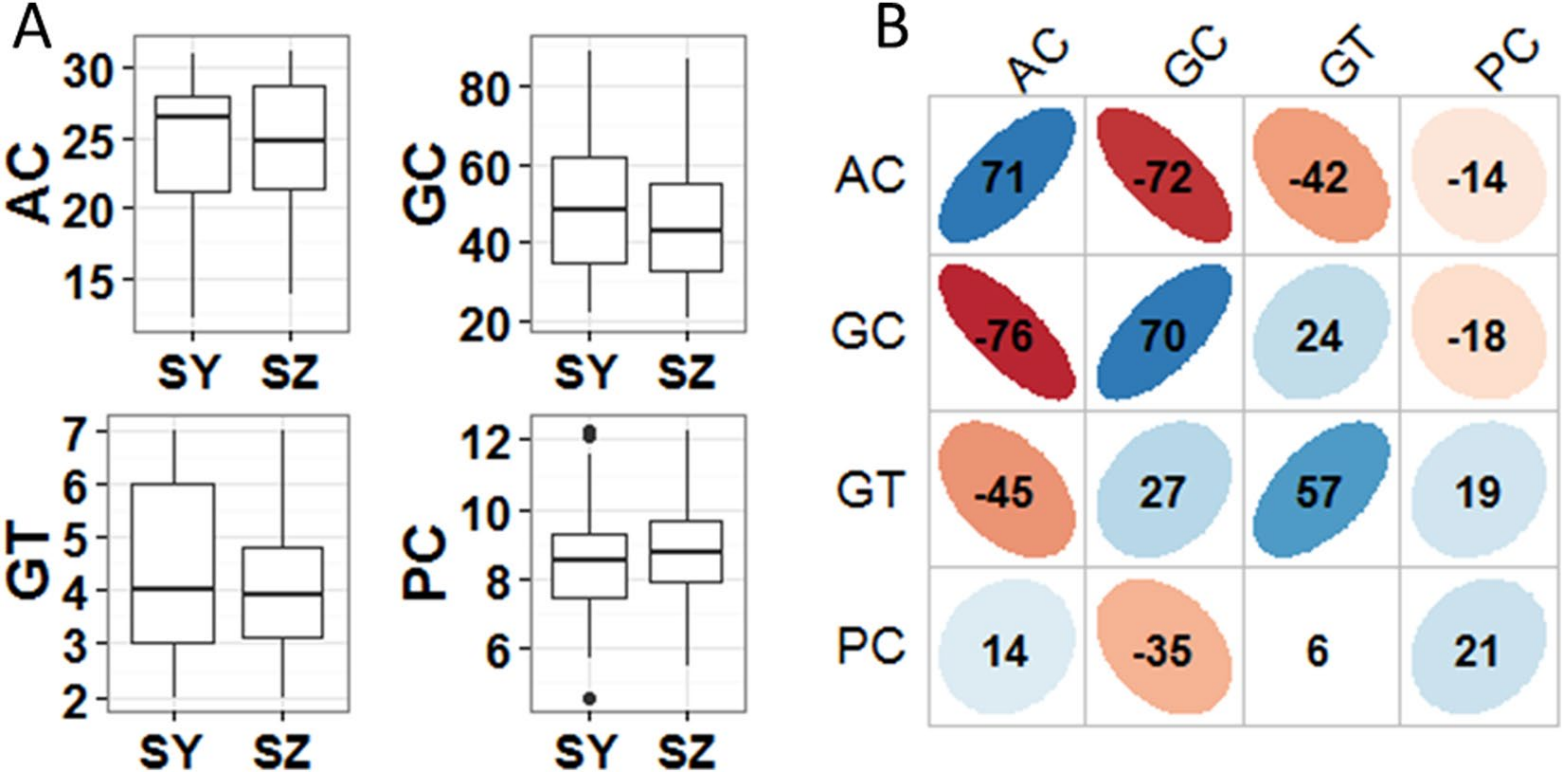
(**A**) Box plots of four investigated traits in two environments. SY: Sanya. SZ: Shenzhen. AC: Apparent Amylose Content. GC: Gel Consistency. GT: Gelatinization Temperature. PC: Protein Content (**B**) Correlations between four evaluated traits in SY (upper triangular) and SZ (lower triangular). The values were correlation coefficients (r) multiplied by 100. The values on principal diagonal indicated correlations between SY and SZ. The areas and colors of ellipses showed the absolute value of corresponding r. Right and left oblique ellipses indicated positive and negative correlations, respectively. The values without glyphs indicated insignificant at 0.05.

Large variations were observed for all investigated traits in both SY and SZ (Fig. 2A). In SY, AC of the evaluated accessions ranged from 12.1% to 31.1% with a mean of 24.6%. GC varied from 21.9 mm to 89.0 mm with a mean of 50.0 mm. GT was averaged at 4.3 ranging from 2.0 to 7.0. PC ranged from 4.6% to 12.3% with an average of 8.5%. In SZ, AC ranged from 13.7% to 31.3% with a mean of 24.6%. GC varied from 21.0 mm to 87.0 mm with a mean of 45.6 mm. GT was averaged at 4.0 ranging from 2.0 to 7.0. PC ranged from 5.5% to 12.3% with an average of 8.8%. The phenotypic pair-wise correlations were almost similar in both two environments (Fig. 2B). AC was negatively correlated with GC and GT with correlation coefficients (r) of −0.72 (−0.76) and −0.42 (−0.45) in SY (SZ), respectively. Significant positive but weak correlation was observed between GC and GT in SY (r = 0.24) and SZ (r = 0.27). PC was significantly weakly correlated with GC with r being −0.18 and −0.35 in SY and SZ, respectively. The phenotypic correlations between SY and SZ ranged from 0.21 for PC to 0.71 for AC (Fig. 2B).

### Identification of QTL by GWAS

**Figure 3:**
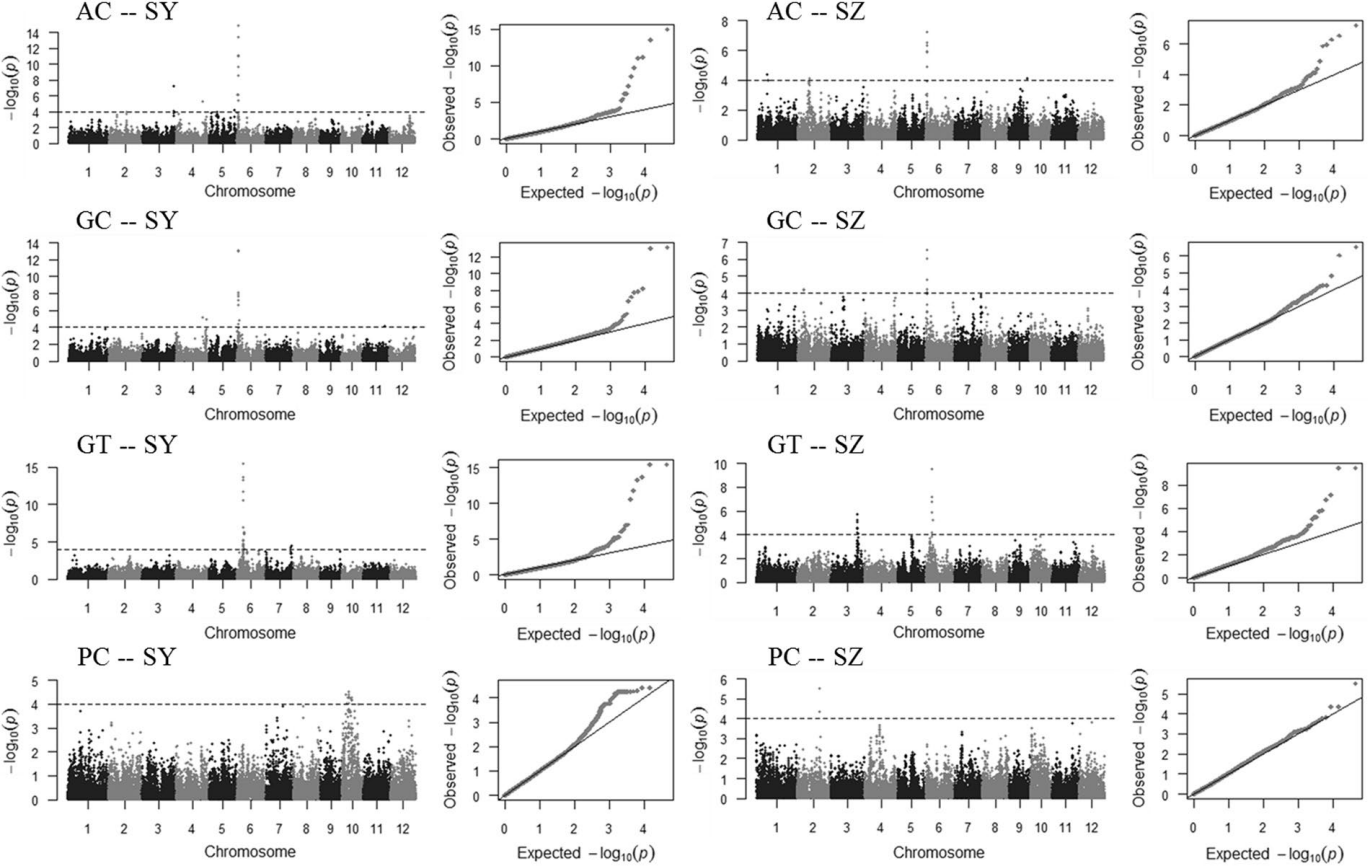
Manhattan and Q-Q plots of GWAS for each trait evaluated in SY and SZ. SY: Sanya. SZ: Shenzhen. AC: Apparent Amylose Content. GC: Gel Consistency. GT: Gelatinization Temperature. PC: Protein Content.

In total, 96 marker-trait associations were detected for the four investigated traits in SY and SZ. For each trait, by delineating significant SNPs on the same chromosome with LD higher than 0.31 into a single QTL, finally 19 QTL were identified (Table 1 and Fig. 3).

**Table 1:** QTL identified by GWAS for AC, GC, GT and PC in SY and SZ.

QTL	Env	Peak	Alleles^a^	MAF^b^	p	Effect^c^	R^2^ (%)^d^	Gene/QTL
*qAC1*	SZ	S1_10888508	C/T	0.47	4.10E-05	−3.5	8.8	*qAC1b*
*qAC2*.*1*	SZ	S2_12269764	C/T	0.24	7.50E-05	−7.1	8.5	
*qAC2*.*2*	SY	S2_19252894	T/G	0.07	9.90E-05	−3.6	6.5	*OsBEIIb*
*qAC3*	SY	S3_33525313	G/A	0.08	5.90E-08	−5.6	12.9	*qAC3*
*qAC4*	SY	S4_28862203	C/T	0.14	5.40E-06	−5.4	8.9	
*qAC5*	SY	S5_27617633	G/T	0.05	6.20E-05	−4.1	7.0	
*qAC6*	SY	S6_1746440	G/A	0.23	1.10E-15	−8.1	31.6	*Wx*
	SZ	S6_1746440	G/A	0.24	5.90E-08	−6.9	16.3	
*qAC9*	SZ	S9_20798975	C/T	0.25	7.30E-05	−6.9	8.3	*qAC-9b (9)*
*qGC2*	SZ	S2_6132333	C/A	0.05	5.70E-05	19.3	8.2	*qGC-2a*
*qGC4*	SY	S4_28862203	C/T	0.14	7.50E-06	22.4	8.7	
*qGC6*	SY	S6_1662107	C/T	0.23	7.00E-14	29.4	26.2	*Wx*
	SZ	S6_1616444	G/A	0.24	2.90E-07	20.0	14.3	
*qGC11*	SY	S11_24266777	A/T	0.13	7.80E-05	16.6	6.7	
*qGC12*	SY	S12_25629093	A/G	0.08	8.50E-05	15.3	6.9	
*qGT3*	SZ	S3_29474609	T/C	0.49	8.70E-05	−0.8	11.9	
*qGT6*	SY	S6_6752888	C/T	0.31	2.56E-16	2.8	41.0	*ALK*
	SZ	S6_6752888	C/T	0.32	6.43E-10	1.2	16.5	
*qGT7*	SY	S7_27788464	C/G	0.12	3.60E-05	−1.5	7.5	
*qPC2*	SZ	S2_24197424	G/C	0.07	3.00E-06	1.6	7.6	
*qPC10*.*1*	SY	S10_7659738	C/T	0.07	2.90E-05	−2.3	8.1	*qPC10*
*qPC10*.*2*	SY	S10_17723490	T/C	0.29	1.00E-04	−1.4	6.7	

^a^Major/Minor allele.

^b^MAF: Minor allele frequency.

^c^Effect: Allele effect with respect to the minor allele.

^d^R^2^ (%): Phenotypic variance explained.

For AC, eight QTL were detected in two environments. Four QTL were identified only in SY including *qAC2*.*2*, *qAC3*, *qAC4* and *qAC5*, which accounted for 6.5%, 12.9%, 8.9% and 7.0% of the phenotypic variance, respectively. Three QTL (*qAC1*, *qAC2*.*1* and *qAC9*) were detected only in SZ explaining 8.8%, 8.5% and 8.3% of the phenotypic variance, respectively. *qAC6* was identified in both SY and SZ, and accounted for 31.6% and 16.3% of the phenotypic variance, respectively (Table 1 and Fig. 3).

Five QTL for GC were identified. These included *qGC4*, *qGC11* and *qGC12*, detected only in SY which explained 8.7%, 6.7% and 6.9% of the phenotypic variance, respectively, and *qGC2* detected only in SZ that accounted for 8.2% of the phenotypic variance. In both SY and SZ, *qGC6* was commonly identified and explained 26.2% and 14.3% of the phenotypic variance, respectively (Table 1 and Fig. 3).

For GT, three QTL were detected. *qGT7* was identified only in SY and explained 7.5% of the phenotypic variance. *qGT3* was detected only in SZ that accounted for 11.9% of the phenotypic variance. *qGT6* was identified in both SY and SZ and explained 41.0% and 16.5% of the phenotypic variance, respectively (Table 1 and Fig. 3).

Three QTL affecting PC were detected. These included *qPC10*.*1* and *qPC10*.*2* identified in SY which explained 8.1% and 6.7% of the phenotypic variance, and *qPC2* identified in SZ that accounted for 7.6% of the phenotypic variance (Table 1 and Fig. 3).

### Candidate genes for important QTL

**Figure 4:**
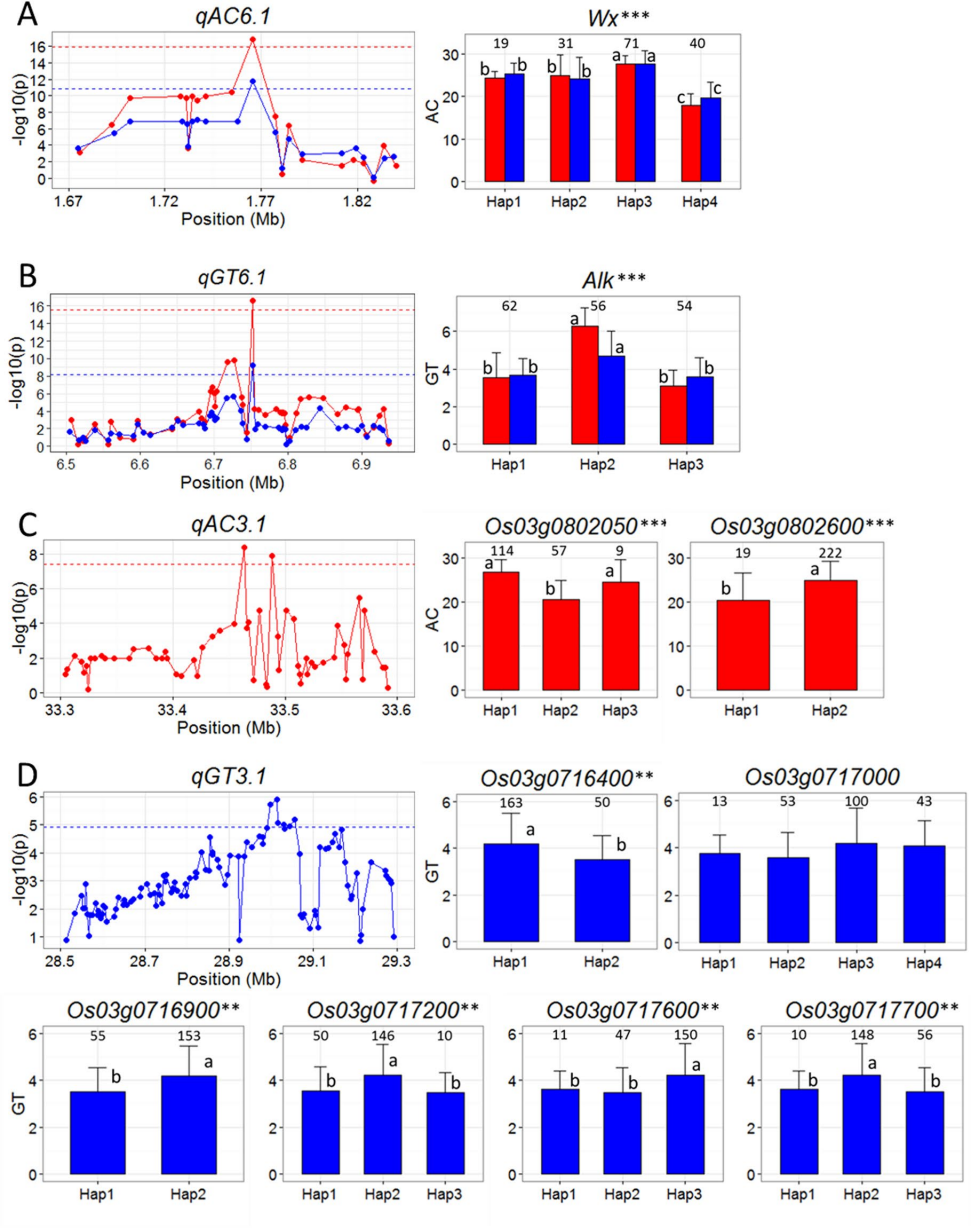
(**A**–**D**) Gene-based association and haplotypes analysis of targeted genes of related QTL including *qAC6* (**A**), *qGT6* (**B**) *qAC3* (**C**) and *qGT3* (**D**). Each point was a gene indicated by one SNPs having largest –log10(p) value. Dash line showed the threshold to determine significant SNP. The ** and *** suggested significance of ANOVA at p < 0.01 and p < 0.001, respectively. The letter on histogram (a and b) indicated multiple comparisons results at the significant level 0.01. The value on the histogram was the number of individuals of each haplotype. Red and blue color indicated Sanya and Shenzhen environments, respectively.

Supplementary Table S1–S4 showed the detailed gene-based association analysis results of the four important QTL regions including *qAC6*, *qGT6*, *qAC3* and *qGT3*. The nine candidate genes were shortlisted based on the haplotype analyses (Fig. 4 and Supplementary Table S5).

**Figure 5:**
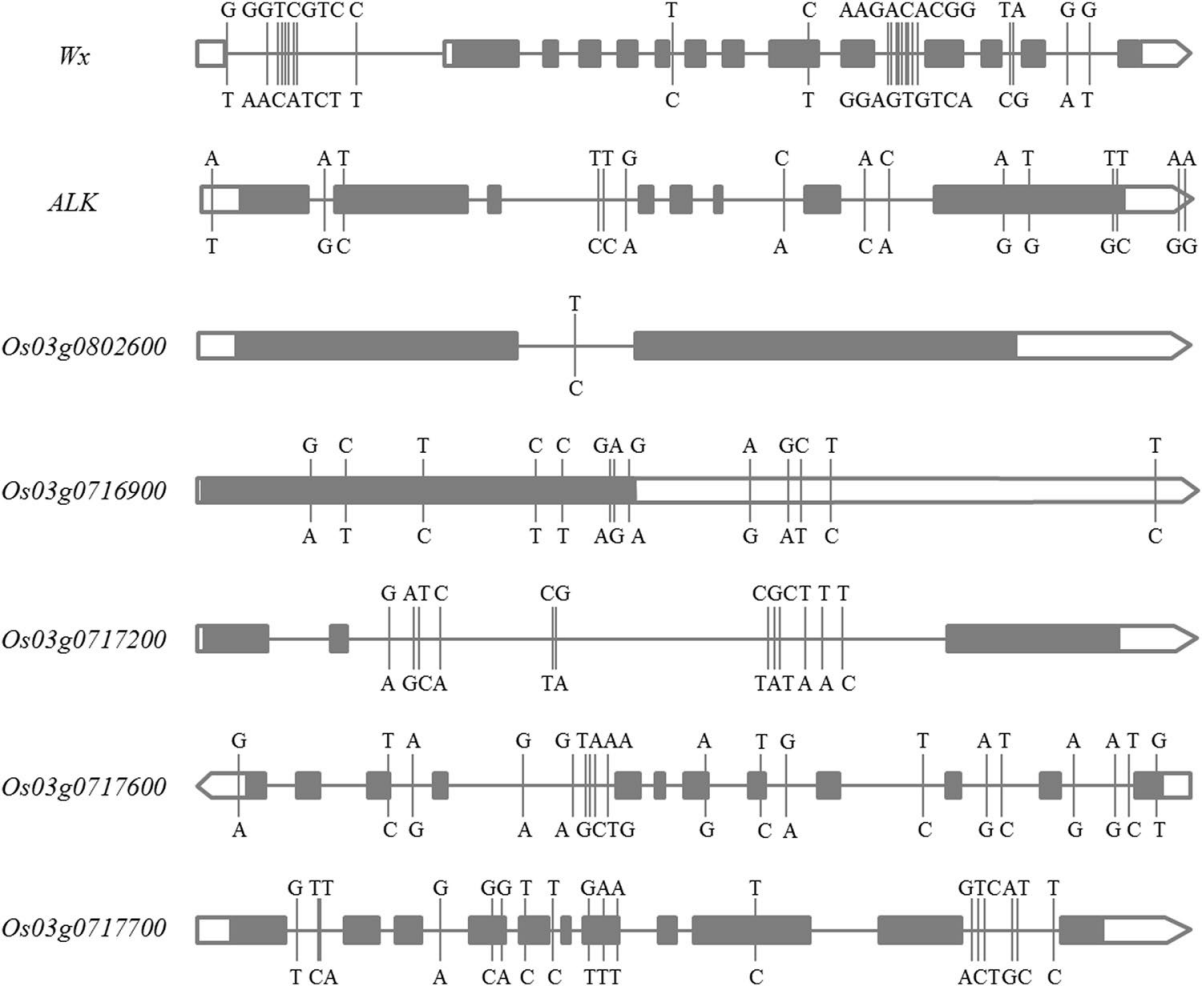
Illustration of gene structure and location of SNPs used in haplotype analysis of seven candidate genes. The alleles above (under) the gene structure could increase (decrease) trait values.

In the region from 1.67 to 1.84 Mb on chromosome 6 harboring *qAC6*, 143 SNPs of 21 genes were used for association analysis. The peak SNP was S6_1765761 with −log10(p) of 16.9 and 11.8 in SY and SZ, respectively. The well-known gene, *Wx* (*Os06g0133000*)^33^ was found to harbor all SNPs with −log10(p) above the threshold (15.9 and 10.8 in SY and SZ, respectively) (Fig. 4A and Supplementary Table S1). In total, 24 SNPs within the *Wx* locus were used for haplotype analysis. Of these SNPs, only a synonymous SNP at the 11^th^ position (S6_1768724) of the *Wx* gene locates in the exon, while all other SNPs locate in the introns (Fig. 5). Four major haplotypes of *Wx* were detected in the investigated panel. Hap3 had highest AC of 27.6% (27.7%) while Hap4 had lowest AC of 18.0% (19.8%) in SY (SZ) (Table S5).

In the region from 6.50 to 6.95 Mb on chromosome 6 harboring *qGT6*, 1,339 SNPs of 53 genes were used for association analyses. The peak SNP was S6_6752888 with −log10(p) of 15.59 and 9.19 in SY and SZ, respectively. Again, all SNPs with −log10(p) above the threshold (14.59 and 8.19 in SY and SZ, respectively) were found to locate in the well-known gene, *ALK* (*Os06g0229800*)^34,35^ (Fig. 4B and Supplementary Table S2), indicating *ALK* was *qGT6*. In total, 15 SNPs within the *ALK* locus were used to analyze haplotypes. These included three SNPs in the 5′UTR and 3′UTR of the gene, two missense mutations in the exons, and the remaining SNPs occurred in the introns (Fig. 5). We noted that the G/A mutation at 10^th^ position of the locus resulted in an amino acid change from Gly to Ser, and the T/C mutation of 13^th^ position changed Phe to Leu. Three major haplotypes of *ALK* were detected in the evaluated panel with Hap2 having significantly higher GT (6.3 ± 1.0 and 4.7 ± 1.3 in SY and SZ) than Hap1 (3.6 ± 1.3 and 3.7 ± 0.9) and Hap3 (3.1 ± 0.8 and 3.6 ± 1.0) (Table S5).

For *qAC3* in the region of 33.3 – 33.6 Mb on chromosome 3, 855 SNPs of 59 genes were used for association analysis. The peak SNP was S3_33463370 (−log10(p) = 8.39). SNPs with −log10(p) above the threshold (7.39) centered around two candidate genes, *Os03g0802050* and *Os03g0802600* (Fig. 4C and Supplementary Table S3). *Os03g0802050* is a gene of unknown function and its gene structure is absent in the RAP-DB. We used 12 SNPs within *Os03g0802050* and detected three haplotypes of in the panel. Hap1 and Hap3 had significantly higher AC than Hap2. *Os03g0802600* is a putative ATPase with a single SNP (S3_33488536) located in the intron of the gene (Fig. 5) with the Hap2 associated with significantly higher AC.

In the region from 28.50 to 29.30 Mb on chromosome 3 harboring *qGT3*, 2,351 SNPs of 107 genes were used for association analysis. The peak SNP was S3_29013799 with –log10(p) being 5.92. Six genes harboring SNPs with –log10(p) larger than the threshold (4.92) were identified, including *Os03g0716400*, *Os03g0716900*, *Os03g0717000*, *Os03g0717200*, *Os03g0717600* and *Os03g0717700* (Fig. 4D and Supplementary Table S4). *Os03g0716400* is a gene of unknown function without functional annotation and gene structure available in RAP-DB. Two major haplotypes consisting of eight SNPs within *Os03g0716400* were detected in the rice panel with Hap1 associated with significantly higher GT. *Os03g0716900* is a hypothetical gene of unknown function. There were 13 SNPs in this gene, including eight nonsynonymous SNPs in its exons that result in amino acids changes, and five SNPs in 3′UTR of the gene (Fig. 5). We detected two haplotypes at *Os03g0716900* in the rice panel with Hap2 having significantly higher GT than Hap1. *Os03g0717000* encodes TMK protein precursor. We identified four haplotypes of this gene in the panel but no significant differences for GT were detected among the haplotypes. *Os03g0717200* encodes a putative cytochrome b561 family protein. There were 12 SNPs all within the introns of this gene (Fig. 5). We identified three haplotypes of this gene in the panel. Hap2 had significantly higher GT than the other two haplotypes. *Os03g0717600* encodes a putative zinc finger (C2H2-type matrin domain containing) protein. There were 19 SNPs within this gene. Three of these SNPs occurred in the exons with a synonymous one plus two nonsynonymous ones causing an amino acid change of from Thr to Ile and another one from Arg into His. The remaining 16 SNPs occurred either in UTR or introns of the gene (Fig. 5). We detected three haplotypes at this gene with Hap3 associated with higher GT. The last candidate of *qGT3*, *Os03g0717700*, encodes a putative histidine kinase. We identified three haplotypes consisting of 18 SNPs in this gene in the rice panel. Seven of these SNPs located in the exons with 5 nonsynonymous ones while the remaining 14 SNPs occurred in the introns (Fig. 5). Hap2 had markedly higher GT than Hap1 and Hap3 (Fig. 4D and Table S5).

## Discussion

Despite tremendous efforts in the past two decades, resolving individual QTL into their causative genes has been a great challenge in dissecting complex traits at the molecular level. In this respect, QTL mapping using diverse panel populations and GWAS has a much higher resolution than the approach by linkage mapping because mapping populations consisting of random rice accessions used in GWAS have much higher recombination accumulated during their evolutionary history. Indeed, most QTL detected in this study were resolved to relatively few candidate genes, and many of them were adjacent to known SSRGs and/or previously identified QTL for related traits. Four QTL for AC, GC and GT identified in this study were close to the known SSRGs. Of these, *qAC6* and *qGC6* were the most important ones detected in the same chromosome region in both environments. This QTL region corresponded to the *Wx* gene (1,765,622 – 1,770,656) encoding GBSSI, a key enzyme for the elongation of amylose chains in amylose biosynthesis well demonstrated previously^1,33,36–38^. *qAC2*.*2* detected in SY was close to *OsBEIIb* (19,355,790 – 19,367,127) encoding a kind of starch branching enzyme. One SNP causing a G/C transversion of *OsBEIIb* was reported to associate with AC^39^. *qGT6*, identified in both SY and SZ, was a major QTL affecting GT. It corresponded to *ALK* encoding SSIIa that mainly affects amylopectin chain-length distribution and alkali disintegration of rice grains^34,40^. *ALK* has been demonstrated to be a major gene affecting GT^1,38,41,42^.

Three other QTL for AC, GC, and PC were adjacent to previously reported QTL for related traits. *qAC1* identified in SZ was located in the QTL region flanked by RM572 – RM449 affecting AC reported previously^16^. *qAC3* detected in SY was equivalent to a QTL of the same name flanked by SSR markers RM416 and RM570 reported by Ebadi, *et al*.^43^. *qAC9* was located in the region of *qAC-9b* flanked by C609 – C506 detected by Wan, *et al*.^44^. *qGC2* was mapped very closely to a QTL region flanked by R712-G227 affecting GC reported by Li, *et al*.^45^. *qPC10*.*1* detected in SY was in approximate vicinity with *qPC10* flanked by RM216 – RM467 detected by Leng, *et al*.^46^. These results indicated that the false positives of the QTL identified in this study were very few, if any. The remaining ten QTL identified in this study, including three QTL for AC (*qAC2*.*1*, *qAC4* and *qAC5*), three for GC (*qGC4*, *qGC11* and *qGC12*), two for GT (*qGT3* and *qGT7*) and two (*qPC2* and *qPC10*.*2*) for PC, were new ones not reported previously. This result would imply that there are many more genes in the rice genome that may have contributed to the tremendous variations for the grain quality traits observed in in the panel.

Our results clearly showed that four large-effect QTL (*qAC6*, *qGT6*, *qAC3* and *qGT3*) detected in this study were all previously reported major genes/QTL affecting rice grain quality traits. The candidate gene of *qAC6* was *Wx* ^33^. It’s reported that *Wx* ^*a*^ and *Wx* ^*b*^ had a G/T mutation at 5′ splicing site of intron 1 which caused the inefficiency of GBSS at the posttranscriptional level, while *Wx* ^*a*^ had higher AC than *Wx* ^*b*^ ^47–49^. Here, we found that a SNP at S6_1765761 (the 1^st^ SNP of the haplotype) was the key mutation that resulted in the lowest AC of Hap4 with nucleotide T when compared the other three haplotypes with nucleotide G. Further, we also observed that Hap1 and Hap2 had lower AC than Hap3. Apparently, other mutations in introns of *Wx* may have also affected its functionality, even though it remains unknown through what unknown mechanism(s) (Fig. 4A and Supplementary Table S5). The candidate gene of *qGT6* was *ALK* ^34^. Bao, *et al*.^3^ reported that the GC/TT polymorphism at 4229/4330 bp of *ALK* was strongly associated with GT variation. Here, we found that two SNPs at S6_6752887 and S6_6752888 in the exon 8 (the 12^th^ and 13^th^ of the haplotype) were the same GC/TT polymorphism sites which caused higher GT of Hap2. Hap1 and Hap3, which differ at a single SNP at S6_6752357 (the 10^th^ SNP of the haplotype) showed no significant differences for GT. This result suggested that this non*-*synonymous mutation at S6_6752357 may not necessarily affect the function of *ALK* (Fig. 4B and Supplementary Table S5).

The above results indicated that gene-based association analysis combining haplotype analysis is an effective way to identify candidate genes for large-effect QTL. We applied this approach to two other large-effect QTL. The first one was *qAC3* which explained 12.9% of the phenotypic variance of AC in SY. Ebadi, *et al*.^43^ also identified a QTL for AC flanked by RM416 and RM570 in this region. Through gene-based analysis, we were able to shortlist this QTL to two candidate genes (*Os03g0802050* and *Os03g0802600*). In the case of *Os03g0802050*, the information for functional annotation and gene structure was not available, so that we could not infer the functional variations based on the SNPs locations in the gene. In the case of *Os03g0802600* which encodes a putative ATPase, we found a single SNP (S3_33488536) in the intron of the gene was responsible for the phenotypic difference between the two haplotypes (Fig. 5), but the mechanism(s) for why Hap2 (T) had higher AC than Hap1 remains unclear (Fig. 4C).

Another case was *qGT3* detected in SZ which accounted for 11.9% of the phenotypic variance. Significant differences in GT were detected between haplotypes of five candidate genes (*Os03g0716400*, *Os03g0716900*, *Os03g0717200*, *Os03g0717600* and *Os03g0717700*), and in all the five cases, significantly increased GT was associated with the major allele(s) (Fig. 4D and Supplementary Table S5), as originally detected in the peak SNP (Table 1). Although, the haplotype differences were insignificant for *Os03g0717000* based on ANOVA, the p value was marginal of 0.0517 (Supplementary Table S5), indicating the weak association of this gene with GT when compared with other candidates. For *Os03g0716900*, Hap2 had significantly higher GT than Hap1, which suggested that the eight missense mutations clustered in the exon affect its function. For *Os03g0717600*, Hap3 differs from Hap1 and Hap2 at two missense SNPs at S3_29042521 and S3_29042839 (the 10^th^ and 11^th^ SNP of haplotype) in exons 6 and 5. Apparently, these two nonsynonymous SNPs were responsible for the phenotypic differences for GT between the haplotypes. In the case of *Os03g0717700*, Hap2 had markedly higher GT than Hap1 and Hap3. The five missense mutations at S3_29054380, S3_29054799, S3_29054902, S3_29054985 and S3_29055829 in exons 5, 7 and 9 of this gene were potentially responsible for the QTL (Fig. 4D and Supplementary Table S5). Taken together, additional evidence from gene knockout or gene knockdown by genetic transformation experiments is required to determine which one or ones of these candidate genes are the real causal one for *qGT3*.

Overall, we identified seven candidates for the two new QTL affecting AC and GT. We realize that the functional inferences of causal genes for the identified QTL based on annotations of gene function and structure may not be sufficient. Currently, further functional validations for these candidates by genetic transformation are in progress to validate the functionalities of the candidate genes on AC and GT. Nevertheless, the current results we presented here provided useful information for genetic validation of the identified QTL candidates and for marker-assisted modification of rice grain quality traits in future breeding.

## Conclusion

Considerable genetic variations for four grain quality traits, AC, GC, GT and PC were observed in the current panel. Through GWAS, 19 QTL for the investigated traits were identified. Among them, four QTL were close to SSRGs and five QTL were adjacent to previously identified QTL for related traits. The remaining 10 QTL (three for AC, three for GC, two for GT and two for PC) were novel ones. Nine candidate genes of four important QTL were determined by gene-based association and haplotype analyses, including two known genes (*Wx* and *ALK*) and seven novels. These newly identified candidate genes affecting rice grain AC and GT provide valuable information for future functional characterization of these candidates and for MAS-based breeding for improving rice grain ECQ.

## Electronic supplementary material

Supplementary Dataset 1
